# Trends and determinants of exclusive and predominant breastfeeding practices for two decades (2000–2019) in Ethiopia

**DOI:** 10.3389/fnut.2025.1516547

**Published:** 2025-01-22

**Authors:** Hailemariam Mamo Hassen

**Affiliations:** Department of Public Health, Dire Dawa University, Dire Dawa, Ethiopia

**Keywords:** exclusive breastfeeding, predominant breastfeeding, disparities, trend analysis, Ethiopia

## Abstract

**Background:**

Ethiopia has had a long-standing national commitment to improving child health. However, evidence on trends in breastfeeding has remained fragmented, and there is a paucity of information on the impacts of breastfeeding policy on breast feeding practices and associated factors influencing it. This study examined trends and determinants of exclusive and predominant breastfeeding in the last two decades.

**Methods:**

The study employed a retrospective observational design using Ethiopian Demographic and Health Surveys (EDHS2000-2019) dataset for children aged <6 months and their mothers. Data analyses were performed via SPSS version 25. Trend analysis and multivariable logistic regression analysis were used.

**Results:**

Exclusive and predominant breastfeeding practices have fluctuated inconsistently over the past two decades. Exclusive breastfeeding increased from 59.96% in 2000 to 66.01% in 2016 and then decreased to 59.86% in 2019. Predominant breastfeeding decreased from 40.04% in 2000 to 32.95% in 2016 and increased to 39.43% in 2019. Regional state, place of residence, and religion were significantly (*p* < 0.001) associated with the likelihood of practicing exclusive or predominant breastfeeding.

**Conclusion:**

These inconsistent trends and the complex interplay of various factors suggest the limited success of previous policies and strategies and highlight the need for further investigation and revisiting current policies for a more nuanced and targeted approach in future interventions.

## 1 Introduction

Breastfeeding is a cornerstone of child health, offering essential nutrients and immune system support for optimal growth and development (1). Breast milk provides bioactive nutrients and antibodies that bolster a child's immune system and growth (2, 3), promote growth, and facilitate tissue repair (3). Breastfeeding also contributes to preventing infectious diseases, gastroenteritis, respiratory disease, childhood diabetes, obesity, and dental disease in children (4). It positively affects the intelligence quotient (IQ) and educational and behavioral outcomes of children (5). Breastfeeding mothers benefit from decreased risks of breast and ovarian cancer and diabetes (6). Compared with non-breastfed children, breastfed children have a reduced risk of acute and chronic illness and improved cognitive outcomes (5), resulting in greater educational achievement and earning potential. Hence, breastfeeding is a critical component of infant and child health, even for mothers (7). The World Health Organization and UNICEF recommend starting breastfeeding within an hour of birth, exclusively nursing for the first 6 months, and continuing breastfeeding for at least 2 years (8) However, challenges persist, particularly in ensuring consistent and equitable breastfeeding, early breastfeeding initiation, exclusive breastfeeding and predominant breastfeeding (9). Exclusive breastfeeding refers to infants receiving only breast milk with no other liquids or solids, while predominant breastfeeding means breast milk is the primary source of nutrition for infants under 6 months (10, 11). Disparities have continued for these practices despite global, national, and local efforts and interventions (12, 13).

For the last five decades, Ethiopia has actively promoted optimal breastfeeding practices (13–17). This dedication reflects a long-standing national commitment to improving child health. Improved access to and use of healthcare has contributed to a rapid decline in under-five mortality rates in Ethiopia (18). A recent study found that the prevalence of under-five mortality in the country was 5.9% (59 deaths per 1,000 live births) (19). This represents significant progress in addressing child health challenges in Ethiopia. The roots of this focus likely lie in the late 1970s and 1980s (20). During this period, a global movement to improve infant and young child feeding practices, including breastfeeding promotion, gained momentum. Ethiopia, which is a significant challenge with respect to childhood mortality rates, highlights the well-documented health benefits of breastfeeding as a potential solution (13–17). This recognition likely sets the stage for the ongoing national commitment to promoting these practices reflected in various national policies and strategies (9, 21, 22). The National Strategy for Infant and Young Child Feeding in Ethiopia aims to improve children's health through better feeding practices (21). It sets national standards for breastfeeding and complementary feeding, emphasizing early breastfeeding initiation and exclusive and predominant breastfeeding for the first 6 months. The National Adolescents, Maternal, Infant, and Young Child Nutrition Guideline (2016–2020) is another policy document in Ethiopia focused on improving nutrition, including breastfeeding (9). Recently, the National Infant and Young Child Feeding in Emergency (IYCF-E) guidelines were released, providing practical guidance for ensuring appropriate infant and young child feeding in emergency response (22).

Multiple factors influence breastfeeding practices beyond a mother's control (12). Exclusive and predominant breastfeeding at the population level can be improved rapidly through multilevel and multicomponent interventions across the socioecological model and settings (1). Such interventions must be guided by research findings for well-informed policies, strategies and initiatives. One such approach is the World Health Organization (WHO) and UNICEF's Baby-Friendly Hospital Initiative (BFHI), which promotes optimal breastfeeding practices by encouraging facilities to implement the Ten Steps to Successful Breastfeeding (23). Ethiopia is implementing the Baby-Friendly Hospital Initiative (BFHI) as part of its multisector National Nutrition Programs to improve child nutrition (8, 24). While positive developments in tackling malnutrition have occurred over the last 10 years, the initial levels were so severe that the country required substantial investment in nutritional programs (24).

Ethiopia's diverse sociocultural landscape and variations in healthcare access necessitate a nuanced examination of exclusive and predominant breastfeeding (24). The country has made notable strides in terms of maternal and child health (25). For instance, the Under-five mortality in Ethiopia decreased by 60% from 2000 to 2016, falling from 166 to 67 deaths per 1,000 live births (19). While previous studies have explored exclusive and predominant breastfeeding, a comprehensive analysis of time trends and determinants over an extended period, mainly using EDHS data in the Ethiopian context, remains limited (9, 15, 16, 26, 27). As far as the researcher knowledge, there has been no recent study on time series data to understand trends in exclusive and predominant breastfeeding practices. Previous studies have been fragmented, and there is a paucity of holistic evidence on the extent of the impacts of policy and implementation strategies. This lack of clarity is particularly concerning when considering potential disparities in exclusive and predominant breastfeeding across different regions and sociodemographic groups within the country. Investigating prevalence trends and disparities will offer insights into the effectiveness of interventions and guide the development of targeted strategies to improve exclusive and predominant breastfeeding practices. It is also crucial for policymakers, healthcare providers, and organizations working to improve child health in Ethiopia.

This study analyzed data from five Ethiopian demographic and health surveys (EDHSs) (2000–2019) (28–32). Therefore, this study aims to fill this gap by examining time trends, and determinants of exclusive and predominant breastfeeding practices (<6 months) across regions and urban/rural areas over the past two decades.

## 2 Materials and methods

### 2.1 Study design and source of data

The present study employed a retrospective observational design using nationally representative Ethiopian Demographic and Health Survey (EDHS) datasets (28–32). Despite, the limitations of the retrospective observational design, as it does not allow for manipulation of variables or random assignment of participants, such a large nationally representative dataset allows for a robust analysis of trends and patterns in exclusive and predominant breastfeeding practices over time. The data for this study were obtained from the Ethiopian Demographic and Health Survey (EDHS) program, a nationally representative cross-sectional survey conducted periodically by the Ethiopian Central Statistical Agency (CSA) and ICF International with financial support from USAID, UKAID, UNICEF, and other donors (28–32).

The EDHS uses a stratified two-stage cluster sampling design to ensure a representative sample of women aged 15–49 across urban and rural areas of Ethiopia. The most recent survey was conducted in 2019, and previous surveys were completed in 2000, 2005, 2011, and 2016. The DHS interviews women in their households, collecting data on a wide range of topics, including demographics, health, and family planning. The specific modules used for collecting breastfeeding data may vary slightly across survey years, but all EDHS surveys adhere to standardized questionnaires and data collection methods to ensure comparability across time periods. By utilizing data from five consecutive DHS datasets (2000–2019), this study provides a valuable opportunity to examine trends and changes in exclusive and predominant breastfeeding practices in Ethiopia over the last two decades. The population base of the study was children living with their mothers, born at least 6 months of age, and the time immediately before the survey was considered.

### 2.2 Study variables and operational definitions

This paper focused on the prevalence trends and disparities of exclusive breastfeeding and predominant breastfeeding, emphasizing disparities in sociodemographic factors across geographical areas and over time. The prevalence in this study context is the proportion of exclusive breastfeeding and predominant breastfeeding practices among mothers of children aged <6 months in Ethiopia from 2000 to 2019 for each EDHS phase, which is calculated by dividing the number of breastfeeding children aged <6 months by the total number of children aged <6 months who lived with their mothers. The prevalence trend is the change in the prevalence of exclusive breastfeeding and predominant breastfeeding among children aged <6 months from 2000 to 2019. Disparities refer to how exclusive breastfeeding and predominant breastfeeding among children aged <6 months are distributed in a population described by independent variables, mainly time factors every 5 years for 20 years, regional state, place of residence, and other maternal and child sociodemographic variables. Hence, the study included exclusive breastfeeding and predominant breastfeeding (dependent variables) and the characteristics of mothers and their children (independent variables).

### 2.3 Outcome variables

The variables related to breastfeeding practices analyzed in the present study focused on the following:

1. *Exclusive breastfeeding* is the prevalence of children aged <6 months who were still breastfeeding at the time of the survey (status quo) and who did not report introducing other foods or liquids before 6 months of age (whether the mother exclusively breastfed for the first 6 months of the child's life by the recall of the mother).
2. *Predominant breastfeeding* is the prevalence of children aged <6 months who are still breastfeeding and who consume only nonnutritive liquids, such as water and sugar-free drinks, in addition to breast milk, but not other foods or liquids.

### 2.3 The independent variables

The sociodemographic characteristics mainly focused on mothers and their children aged <6 months in Ethiopia. The survey year (time), mother age group, regional state, place of residence, mother's current marital status, mother's education level, religion, mother's employment status, current pregnancy status, place of delivery, antenatal care utilization, postnatal care utilization and number of household members or family size were characteristics considered as independent variables.

### 2.4 Data acquisition and extraction

The data for this study were acquired from the Ethiopian Demographic and Health Survey (EDHS) datasets covering the period from 2000 to 2019 in Ethiopia, which were obtained following formal application to the program Data Archivist (28–32). The EDHS surveys employed rigorous sampling methodologies to ensure national representativeness, and the data were collected through standardized interviews, questionnaires, and measurements. Ethical considerations are paramount in the EDHS data collection process. Hence, the utilization of these datasets for the current research follows the terms and conditions specified by the Demographic and Health Survey Program.

**Figure 1:**
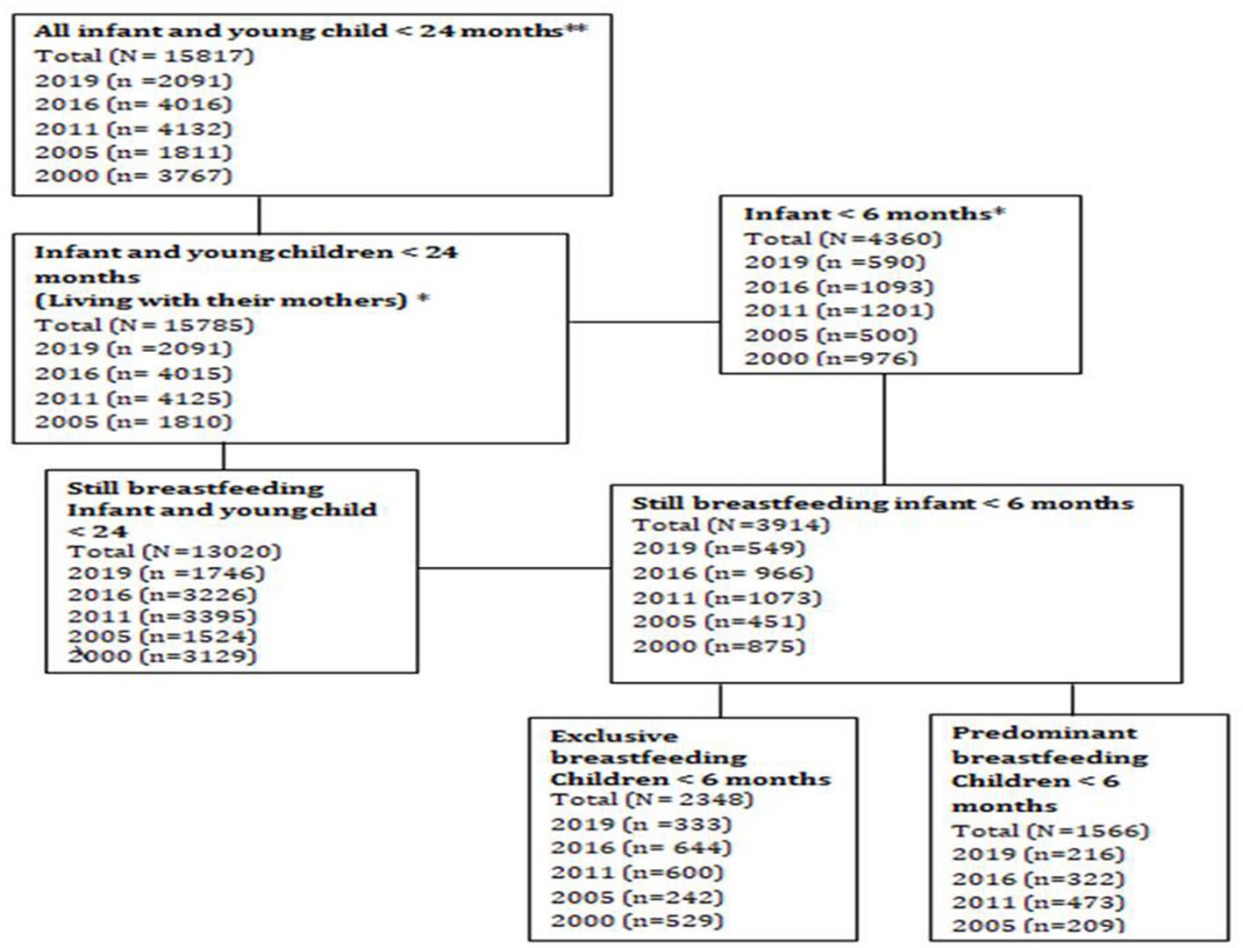
Flow diagram of the extraction of study samples for the analysis of exclusive and predominant breastfeeding practices, Ethiopia 2000–2019. **All infants and young children, including those who died. *All infants and young children living with their mothers, including those who died.

The samples of the analysis were performed from the five consecutive surveys stratifying and extracting the exclusive and predominant breastfeeding practices and the presumed independent variables for children aged <6 months (Figure 1). The first datasets for all infants and young children, including children who died, were taken, and then young children living with their mothers were identified. Next, children aged <6 months and living with their mothers and those who practice exclusive and predominant breastfeeding were identified. Finally, infants and young children living with their mothers, who practice exclusive and predominant breastfeeding, were filtered consecutively (Figure 1).

### 2.5 Data analysis

Data analysis was performed via the Statistical Package for Social Sciences (SPSS) software version 25. Data were weighted for the survey year and mothers' characteristics. The study utilized descriptive statistics to examine the overall prevalence of exclusive and predominant breastfeeding practices over the last five consecutive surveys. Time series analysis was employed to discern temporal patterns and changes (trends) in exclusive and predominant breastfeeding practices. Multivariate logistic regression models were used to identify the factors associated with exclusive and predominant breastfeeding practices, with *p* ≤ 0.05 at the 95% confidence interval. Throughout the analysis, potential confounders were controlled by stratification, restriction, and statistical approaches. The analysis was based on the assumptions of sufficient data, linear relationships between variables (after transformation), the absence of multicollinearity (independent variables are not highly correlated), and the absence of outliers.

## 3 Results

### 3.1 Characteristics of study participants

According to the results of the Pearson chi-square test, children aged <6 months and their mothers were distributed differently across the indicated sociodemographic and related characteristics (*p* < 0.001), with the exception of the mothers' age group (*p* = 0.66) and family size (*p* = 0.467) (Table 1). Approximately 3.59% of the children aged <6 months during the data collection period died. The sociodemographic characteristics of mothers and their children in the five surveys differed significantly (EDHS 2000–2019) (Table 1). The majority of mothers (73.89%) were 20–34 years old, and 94.50% of the mothers surveyed were married during the survey. Proportionally, approximately 58.20% of mothers and their children lived in the four populated regional states, namely, Oromia, Amhara, Tigray, and the former Southern Nations and Nationalities and Peoples (SNNP). Most mothers and their children resided in rural areas, which declined slightly across the five survey years, from 84.13% in 2000 to 78.52% in 2019. Most mothers were uneducated, which declined from 79.46% in 2000 to 48.56% in 2019, and a significant number of mothers did not complete primary education, with the proportion decreasing across the survey years (Table 1).

**Table 1:** Characteristics of mothers and their children aged <6 months in Ethiopia.

**Respondents Characteristics**	**Survey year (2000–2019)**					**Pearson chi-square *P* values**
	**2000**	**2005**	**2011**	**2016**	**2019**	
	***n*** **(%)**	***n*** **(%)**	***n*** **(%)**	***n*** **(%)**	***n*** **(%)**	
	964 (22.94)	594 (11.75)	1158 (27.55)	1033 (24.58)	554 (13.18)	
**Mothers age in 5 years group**						
15–19	96 (9.96)	53 (10.73)	111 (9.59)	88 (8.52)	65 (11.73)	0.066
20–24	257 (26.66)	131 (26.52)	288 (24.87)	262 (25.36)	130 (23.47)	
25–29	259 (26.87)	145 (29.35)	358 (30.92)	294 (28.46)	161 (29.06)	
30–34	190 (19.71)	89 (18.02)	189 (16.32)	213 (20.62)	117 (21.12)	
35–39	124 (12.86)	52 (10.53)	154 (13.30)	143 (13.84)	56 (10.11)	
40–44	35 (3.63)	18 (3.64)	50 (4.32)	30 (2.90)	24 (4.33)	
45–49	3 (0.31)	6 (1.21)	8 (0.69)	3 (0.29)	1 (0.18)	
**Regional states**						
Tigray	98 (10.17)	42 (8.50)	100 (8.64)	115 (11.13)	56 (10.11)	< 0.001
Afar	49 (5.08)	33 (6.68)	118 (10.19)	99 (9.58)	64 (11.55)	
Amhara	136 (14.11)	79 (15.99)	116 (10.02)	99 (9.58)	43 (7.76)	
Oromia	179 (18.57)	92 (18.62)	199 (17.18)	164 (15.88)	72 (13.00)	
Somali	51 (5.29)	28 (5.67)	117 (10.10)	140 (13.55)	58 (10.47)	
Ben-Gumz	92 (9.54)	35 (7.09)	97 (8.38)	86 (8.33)	49 (8.84)	
Former SNNP	148 (15.35)	95 (19.23)	162 (13.99)	115 (11.13)	55 (9.93)	
Gambela	51 (5.29)	25 (5.06)	93 (8.03)	60 (5.81)	49 (8.84)	
Harari	52 (5.39)	32 (6.48)	53 (4.58)	58 (5.61)	43 (7.76)	
Addis Ababa	53 (5.50)	15 (3.04)	36 (3.11)	49 (4.74)	24 (4.33)	
Dire Dawa	55 (5.71)	18 (3.64)	67 (5.79)	48 (4.65)	41 (7.40)	
**Place of residence**						
Urban	153 (15.87)	69 (13.97)	174 (15.03)	202 (19.55)	119 (21.48)	< 0.001
Rural	811 (84.13)	425 (86.03)	984 (84.97)	831 (80.45)	435 (78.52)	
**Mother's current marital status**						
Never married	10 (1.04)	6 (1.21)	6 (0.52)	7 (0.68)	8 (1.44)	< 0.001
Married	911 (94.50)	463 (93.72)	1,047 (90.41)	987 (95.55)	525 (94.77)	
Living together	11 (1.14)	13 (2.63)	57 (4.92)	12 (1.16)	6 (1.08)	
Widowed	8 (0.83)	2 (0.40)	10 (0.86)	3 (0.29)	2 (0.36)	
Divorced	12 (1.24)	5 (1.01)	20 (1.73)	14 (1.36)	7 (1.26)	
Not living together	12 (1.24)	5 (1.01)	18 (1.55)	10 (0.97)	6 (1.08)	
**Mother education attainment**						
No education	766 (79.46)	370 (74.90)	769 (66.41)	601 (58.18)	269 (48.56)	< 0.001
Incomplete primary	106 (11.00)	81 (16.40)	300 (25.91)	263 (25.46)	159 (28.70)	
Complete primary	13 (1.35)	7 (1.42)	21 (1.81)	25 (2.42)	31 (5.60)	
Incomplete Secondary	53 (5.50)	23 (4.66)	42 (3.63)	97 (9.39)	57 (10.29)	
Complete secondary	23 (2.39)	5 (1.01)	7 (0.60)	8 (0.77)	7 (1.26)	
Higher	3 (0.31)	8 (1.62)	19 (1.64)	39 (3.78)	31 (5.60)	
**Religion**						
Muslim	370 (38.38)	183 (37.04)	574 (49.57)	523 (50.63)	296 (53.43)	
Orthodox	422 (43.78)	184 (37.25)	323 (27.89)	316 (30.59)	154 (27.80)	< 0.001
Protestant	139 (14.42)	116 (23.48)	226 (19.52)	170 (16.46)	99 (17.87)	
Catholic	5 (0.52)	2 (0.40)	10 (0.86)	3 (0.29)	2 (0.36)	
Traditional	28 (2.90)	9 (1.82)	11 (0.95)	9 (0.87)	3 (0.54)	
**Place of delivery**						
Home	837 (88.20)	439 (88.69)	992 (85.81)	591 (57.05)	251 (45.23)	< 0.001
Government/public health facilities	104 (10.96)	48 (9.70)	135 (11.68)	398 (38.42)	285 (51.35)	
Private/NGO health facilities	8 (0.84)	8 (1.62)	29 (2.51)	47 (4.54)	19 (3.42)	
**Antenatal care utilization**						
No antenatal visits	648 (67.64)	346 (70.76)	674 (58.20)	330 (31.95)	145 (26.17)	< 0.001
1- or 2-times antenatal visits	149 (15.55)	46 (9.41)	162 (13.99)	179 (17.33)	69 (12.45)	
At least 3-time antenatal visits	161 (16.81)	97 (19.84)	322 (27.81)	524 (50.73)	340 (61.37)	
**Postnatal care utilization**						
No	-	253 (95.47)	960 (96.48)	949 (91.87)	500 (90.25)	< 0.001
Yes	-	12 (4.53)	35 (3.52)	84 (8.13)	54 (9.75)	
**Number of household members/family size**						
1–2	7 (0.73)	1 (0.20)	15 (1.28)	12 (1.15)	3 (0.54)	0.467
3–4	241 (25.00)	134 (27.07)	274 (23.46)	279 (26.72)	145 (25.99)	
5–6	305 (31.64)	163 (32.93)	380 (32.53)	332 (31.80)	186 (33.33)	
7+	411 (42.63)	197 (39.80)	499 (42.72)	421 (40.33)	224 (40.14)	

The proportion of home births in Ethiopia has steadily declined, dropping from 88.20% in 2000 to 45.23% in 2019. The proportion of mothers who did not receive antenatal care declined from 67.64% in 2000 to 26.17% in 2019. Most mothers did not utilize postnatal care. Most of the mothers and their children lived in a family of five or more household members (Table 1).

### 3.2 Trends in exclusive breastfeeding and predominant breastfeeding in Ethiopia

**Figure 2:**
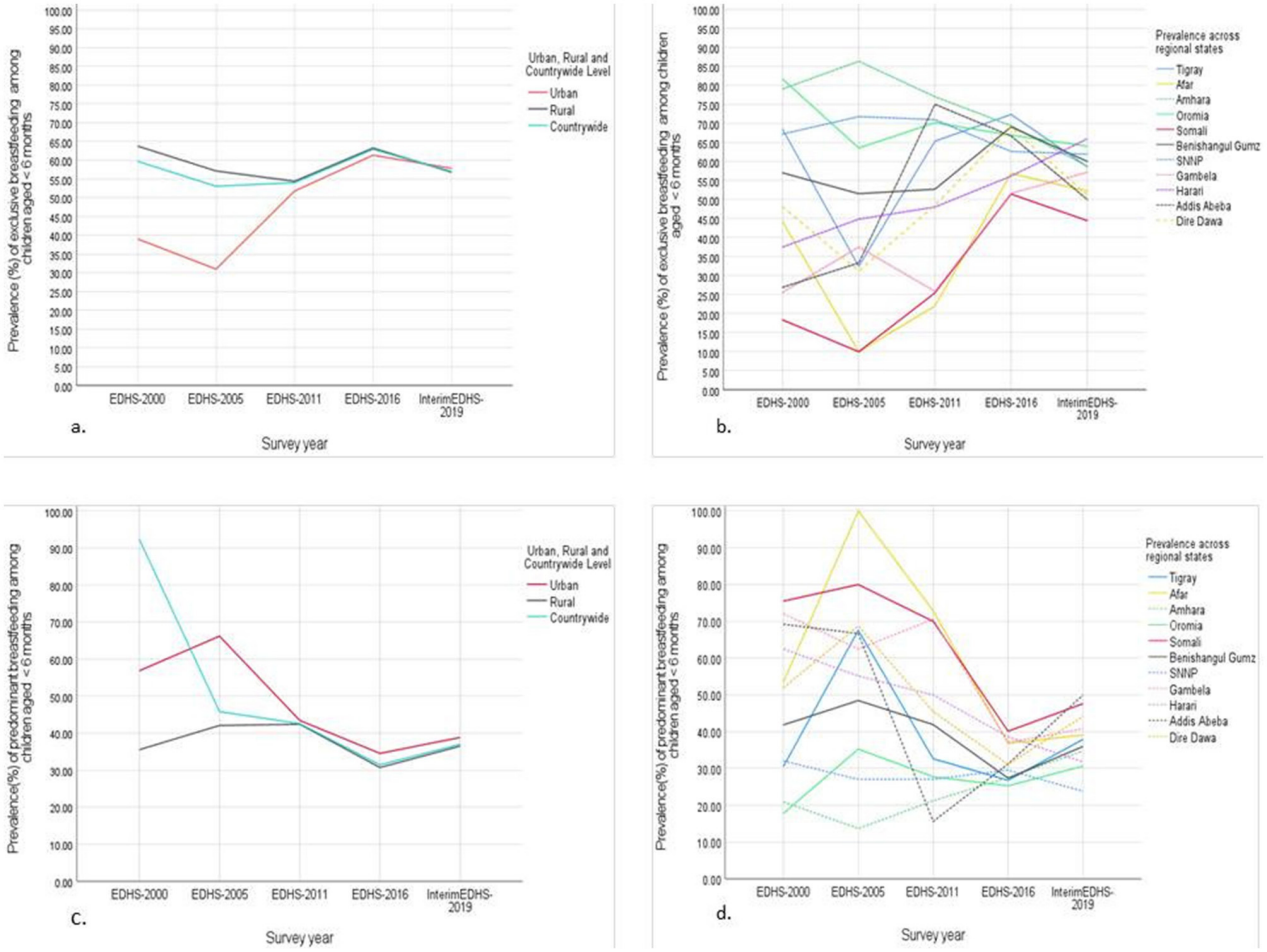
The prevalence trends and disparities of **(A)** exclusive breastfeeding in urban, rural and countrywide level, **(B)** exclusive breastfeeding across regional states level **(C)** predominant breastfeeding in urban, rural and countrywide level and **(D)** predominant breastfeeding across regional states level among Ethiopian children (<6 months) (EDHS, Ethiopian Demographic and Health Survey).

According to the visualization of multiseries line graphs, the pattern of prevalence and trends of exclusive breastfeeding and predominant breastfeeding has fluctuated inconsistently and changed in the last 20 years across states/regions and urban, rural, and countrywide levels (Figure 2). Exclusive breastfeeding increased from 59.96% in 2000 to 66.01% in 2016. However, it then slightly decreased to 59.86% in 2019. Predominant breastfeeding decreased from 40.04% in 2000 to 32.95% in 2016 and slightly increased to 39.43% in 2019, generally declining over the past two decades (Figure 2). These estimates of exclusive breastfeeding and predominant breastfeeding prevalence and changes over time revealed disparities across states/regions, urban areas, rural areas, and countrywide levels.

Children from rural areas were more likely to practice exclusive breastfeeding than those from urban areas were, which declined in 2005 and then increased until 2016 (Figure 2A). Compared with those in rural areas, children in urban areas are more likely to practice predominant breastfeeding, which has continued to decline in both areas since the survey year of 2005 and has slightly increased since 2016 (Figure 2C). In general, the pattern of the prevalence of exclusive breastfeeding (Figure 2B), and predominant breastfeeding was similar across regional states, except for the pattern for Gambella from 2000 to 2011 (Figure 2C).

### 3.3 Factors associated with exclusive and predominant breastfeeding in Ethiopia

A multicollinearity test was performed to assess the degree of correlation among the independent variables to ensure the reliability and interpretability of the model, checking that the variables were not highly correlated. The variance inflation factor (VIF) ranged from 1.286 to 3.704, which was within the acceptable limit. Year of the survey, mothers' age group, regional state, place of residence, marital status, educational attainment, religion, mothers' current working status, delivery location, antenatal and postnatal care utilization, and family size—were examined.

While there seems to be a fluctuation in the prevalence of exclusive and predominant breastfeeding in Ethiopia from 2000 to 2019 across the years, the present study does not show significant associations between exclusive breastfeeding or predominant breastfeeding and the year of the survey using AORs. The study revealed that regional state, place of residence, and maternal religion were significantly (*p* < 0.001) associated with the likelihood of exclusively breastfeeding. Mothers residing in Amhara (AOR: 5.036), Oromia (AOR: 4.418), Tigray (AOR: 2.574), Benishangul-Gumz (AOR: 2.149), former SNNP (AOR: 3.997), Harari (AOR: 1.740), Addis Ababa (AOR: 2.535), and Dire Dawa (AOR: 2.117) have a much greater likelihood of practicing exclusively than mothers in the Somali region (*p* < 0.001).

Similarly, the study revealed a statistically significant association between a regional state and the likelihood of practicing predominant breastfeeding. Mothers residing in Amhara (AOR: 4.479), Oromia (AOR: 4.418), Tigray (AOR: 2.452), Benishangul-Gumz (AOR: 2.020), former SNNP (AOR: 3.204), Harari (AOR: 1.440), Addis Ababa (AOR: 1.593), and Dire Dawa (AOR: 1.559) have a much greater likelihood of practicing predominant breastfeeding than mothers in the Somali region (*p* < 0.01) (Table 2). Mothers between 25 and 29 years old were significantly associated with exclusive breastfeeding; these mothers were somewhat less likely to practice exclusive breastfeeding (AOR: 0.752, *p* = 0.013) and predominant breastfeeding (AOR: 0.778, *p* = 0.03) than mothers aged 15–19 years were. However, there was no statistically significant association for the other age groups. Mothers living in rural areas are significantly more likely to practice exclusive breastfeeding (AOR: 1.407) and predominant breastfeeding (AOR: 1.406) than mothers in urban areas are (*p* < 0.001) (Table 2).

**Table 2:** Prevalence and associated factors of exclusive and predominant breastfeeding (children < 6 months).

**Independent variables**	**Dependent variables**					
	**Exclusive breastfeeding (children**<**6 months)**			**Predominant breastfeeding (children**<**6 months)**		
	**Prevalence (95%CI)**	**AOR (95%CI)**	***P* value**	**Prevalence (95%CI)**	**AOR (95%CI)**	***P* value**
**Survey year**						
2000	59.96 (56.84–63.02)	Ref.		40.04 (36.98–43.16)	Ref.	
2005	54.75 (50.35–59.09)	0.815 (0.656–1.013)	0.065	45.05 (40.71–49.45)	0.813 (0.653–1.011)	0.062
2011	55.22 (52.36–58.06)	0.799 (0.673–0.948)^**^	0.010	43.92 (41.09–46.78)	0.877 (0.739–1.042)	0.136
2016	66.00 (63.08–68.82)	1.174 (0.984–1.402)	0.075	32.95 (30.15–35.85)	1.424 (1.189–1.707)	0.081
2019	59.86 (55.75–63.86)	0.898 (0.730–1.105)	0.310	39.43 (35.43–43.53)	1.100 (0.891–1.358)	0.373
**Mothers age group**						
15–19	62.47 (57.73–67.04)	Ref.		37.53 (32.96–42.27)	Ref.	
20–24	62.43 (59.50–65.30)	0.992 (0.789–1.247)	0.943	37.38 (34.52–40.31)	1.010 (0.801–1.275)	0.931
25–29	55.60 (52.81–58.37)	0.752 (0.601–0.941)^*^	0.013	43.91 (41.14–46.70)	0.778 (0.620–0.976)^*^	0.030
30–34	60.52 (57.12–63.85)	0.931 (0.733–1.182)	0.557	38.24 (34.94–41.63)	0.966 (0.758–1.231)	0.780
35–39	60.30 (56.10–64.38)	0.914 (0.706–1.183)	0.496	38.76 (34.70–42.95)	0.951 (0.732–1.236)	0.709
40–44	56.25 (48.51–63.77)	0.787 (0.547–1.133)	0.198	41.88 (34.43–49.61)	0.831 (0.575–1.202)	0.325
45–49	47.62 (27.73–68.12)	0.498 (0.213–1.162)	0.107	52.38 (31.88–72.27)	0.626 (0.270–1.453)	0.276
**Regional state**						
Somali	39.39 (34.67–44.27)	Ref.		60.10 (55.22–64.84)	Ref.	
Afar	40.55 (35.60–45.64)	1.031 (0.745–1.425)	0.856	58.90 (53.80–63.86)	1.011 (0.765–1.336)	0.940
Amhara	76.68 (72.73–80.31)	5.036 (3.473–7.301)^***^	< 0.001	22.69 (19.10–26.61)	4.479 (3.359–5.973)^***^	< 0.001
Oromiya	72.29 (68.92–75.49)	4.418 (3.282–5.947)^***^	< 0.001	27.00 (23.84–30.36)	3.631 (2.819–4.678)^***^	< 0.001
Tigray	64.81 (60.10–69.30)	2.574 (1.760–3.765)^***^	< 0.001	34.95 (30.47–39.65)	2.452 (1.855–3.241)^***^	< 0.001
Benshangul–Gumz	58.84 (53.72–63.82)	2.149 (1.541–2.996) ^***^	< 0.001	40.33 (35.37–45.45)	2.020 (1.522–2.681)^***^	< 0.001
Former SNNP	69.72 (65.89–73.36)	3.997 (2.801–5.704)^***^	< 0.001	29.76 (26.14–33.58)	3.204 (2.467–4.161)^***^	< 0.001
Gambela	39.22 (33.67–45.00)	1.028 (0.672–1.573)	0.897	59.01 (53.21–64.63)	0.960 (0.710–1.299)	0.793
Harari	51.46 (45.14–57.75)	1.740 (1.209–2.503)^**^	0.003	48.12 (41.84–54.44)	1.440 (1.049–1.977)^*^	0.024
Addis Ababa	53.67 (46.32–60.91)	2.535 (1.584–4.057) ^***^	< 0.001	46.33 (39.09–53.68)	1.593 (1.124–2.259)^**^	0.009
Dire Dawa	53.04 (46.59–59.42)	2.117 (1.465–3.060)^***^	< 0.001	46.52 (40.15–52.98)	1.559 (1.132–2.149)^**^	0.007
**Place of residence**						
Urban	52.57 (48.92–56.21)	Ref.		47.15 (43.52–50.80)	Ref.	
Rural	60.94 (59.32–62.54)	1.407 (1.201–1.648)^***^	< 0.001	38.38 (36.78–39.99)	1.406 (1.199–1.648)^***^	< 0.001
**Mother current marital status**						
Never married	62.16 (46.10–76.40)	Ref.		37.84 (23.60–53.90)	Ref.	
Married	59.47 (57.93–60.99)	1.068 (0.575–1.986)	0.835	39.97 (38.46–41.51)	0.818 (0.428–1.565)	0.544
Living together	62.63 (52.84–71.68)	1.213 (0.582–2.527)	0.606	37.37 (28.32–47.16)	0.911 (0.425–1.950)	0.810
Widowed	56.00 (36.78–73.91)	0.913 (0.340–2.451)	0.857	44.00 (26.09–63.22)	0.707 (0.257–1.943)	0.502
Divorced	62.30 (49.79–73.66)	1.239 (0.556–2.760)	0.600	32.79 (22.01–45.15)	1.089 (0.472–2.514)	0.842
Not living together	53.85 (40.40–66.88)	0.843 (0.373–1.905)	0.681	44.23 (31.35–57.74)	0.699 (0.301–1.621)	0.404
**Mother education attainment**						
No education	59.08 (57.24–60.89)	Ref.		40.28 (38.47–42.11)	Ref.	
Incomplete primary	62.14 (58.97–65.25)	1.129 (0.972–1.311)	0.113	37.31 (34.22–40.48)	1.132 (0.972–1.318)	0.112
Complete primary	61.86 (51.96–71.06)	1.150 (0.763–1.732)	0.505	38.14 (28.94–48.04)	1.072 (0.709–1.622)	0.741
Incomplete secondary	55.31 (49.38–61.13)	0.888 (0.694–1.138)	0.349	44.32 (38.52–50.25)	0.830 (0.648–1.065)	0.143
Complete secondary	42.00 (29.09–55.81)	0.490 (0.281–0.855) ^*^	0.012	58.00 (44.19–70.91)	0.530 (0.307–0.914)^*^	0.023
Higher	65.69 (56.14–74.36)	1.391 (0.921–2.099)	0.116	32.35 (23.87–41.83)	1.357 (0.891–2.067)	0.154
**Religion of mothers**						
Muslim	53.65 (51.44–55.86)	Ref.		45.78 (43.58–48.00)	Ref.	
Catholic	52.17 (32.52–71.31)	1.028 (0.451–2.339)	0.948	43.48 (24.99–63.50)	1.019 (0.445–2.335)	0.964
Protestant	59.74 (56.21–63.19)	1.303 (1.103–1.540) ^**^	0.002	39.60 (36.16–43.13)	1.253 (1.058–1.484) ^**^	0.009
Orthodox	67.71 (65.23–70.12)	1.868 (1.624–2.149)^***^	< 0.001	31.79 (29.40–34.26)	1.727 (1.499–1.991)^***^	< 0.001
Traditional and others	57.89 (44.95–70.06)	1.243 (0.734–2.106)	0.418	38.60 (26.78–51.54)	1.283 (0.749–2.196)	0.364
**Mothers currently working**						
No	59.06 (57.18–60.93)	Ref.		40.37 (38.51–42.25)	Ref.	
Yes	60.53 (57.50–63.49)	1.093 (0.946–1.264)	0.228	38.79 (35.83–41.80)	1.046 (0.903–1.211)	0.552
**Delivery place**						
Home	59.29 (57.56–61.01)	Ref.		40.13 (38.42–41.86)	Ref.	
Public Health Facilities	60.00 (56.89–63.05)	1.019 (0.882–1.176)	0.799	39.28 (36.24–42.38)	1.042 (0.901–1.206)	0.579
Private/NGO Facilities	64.86 (55.69–73.27)	1.206 (0.823–1.766)	0.336	34.23 (25.90–43.38)	1.310 (0.883–1.943)	0.179
**Antenatal care utilization**						
No antenatal visits	59.03 (56.94–61.10)	Ref.		40.97 (38.90–43.06)	Ref.	
1 or 2 times antenatal visits	61.32 (57.40–65.14)	1.099 (0.917–1.316)	0.308	38.51 (34.70–42.44)	1.104 (0.919–1.326)	0.289
At least 3 time antenatal visits	60.60 (58.06–63.09)	1.067 (0.934–1.219)	0.341	39.40 (36.91–41.94)	1.064 (0.930–1.218)	0.366
**Postnatal care utilization with 2 months**						
No	60.33 (58.46–62.18)	Ref.		39.67 (37.82–41.54)	Ref.	
Yes	57.30 (50.10–64.27)	0.955 (0.707–1.288)	0.761	42.70 (35.73–49.90)	0.838 (0.620–1.132)	0.249
**Number of household members/family size**						
1–2	47.37 (32.17–62.95)	Ref.		52.63 (37.05–67.83)	Ref.	
3–4	59.27 (56.31–62.18)	1.579 (0.832–2.996)	0.162	40.63 (37.72–43.59)	1.618 (0.853–3.066)	0.140
5–6	61.49 (58.89–64.05)	1.738 (0.918–3.290)	0.090	37.99 (35.45–40.59)	1.795 (0.949–3.394)	0.072
7+	58.39 (56.07–60.68)	1.515 (0.802–2.862)	0.201	40.58 (38.30–42.90)	1.629 (0.863–3.073)	0.132

^*^p < 0.05, ^**^p < 0.01, ^***^p < 0.001, Ref., reference category.

Statistically significant associations were observed between a mother's religion and the likelihood of practicing exclusive breastfeeding (EBF) and predominantly breastfeeding (Table 2). Compared with Muslim mothers, Protestant mothers (AOR: 1.303, *p* = 0.002) and Orthodox mothers (AOR: 1.868, *p* < 0.001) are significantly more likely to practice EBF. Similarly, protestant (AOR: 1.253, *p* = 0.009) and Orthodox mothers (AOR: 1.727, *p* < 0.001) are significantly more likely to practice predominant breastfeeding than Muslim mothers are. Compared with mothers with no education, mothers with complete secondary education had a significantly lower prevalence of EBF and predominantly breastfeeding (AOR: 0.490, *p* = 0.012). While the present study did not show statistically significant associations with other factors via AORs, there was evidence of disparities across these factors (Table 2).

## 4 Discussion

In the present study, children aged <6 months and their mothers were distributed unevenly across the majority of sociodemographic characteristics, implying that these factors are likely associated with exclusive and predominant breastfeeding practices. The number of home births in Ethiopia has shown a positive trend, declining significantly from 88.20% in 2000 to 45.23% in 2019, implying increased literacy and improved access to and utilization of maternal healthcare services, as depicted in other studies (33, 34). Similarly, the decline in the proportion of mothers who did not utilize antenatal care (ANC) from 67.64% in 2000 to 26.17% in 2019 is a positive development, as other studies confirmed that the utilization of maternal healthcare services in Ethiopia is improving (16, 35). However, a significant concern is that most mothers do not utilize postnatal care. Most of the mothers and their children in Ethiopia lived in families of five or more members.

The WHO and UNICEF recommend that children should only receive breast milk for the first 6 months of life (exclusive breastfeeding) or that they may also receive small amounts of water, oral rehydration solutions (ORSs), or medicines (predominantly breastfeeding) (36, 37). This nationwide population-based study investigated exclusive and predominant breastfeeding practices over the last two decades, indicating the impact of policies and strategies (9, 21, 22). Previous studies were fragmented and focused on specific populations and timeframes, hindering a complete understanding of national prevalence trends through time and disparities (9). The time series analysis revealed that the trends of exclusive and predominant breastfeeding in Ethiopia were inconsistent and fluctuated across different regions over the last 20 years.

The observed trend in exclusive breastfeeding rates—an increase from 2000 to 2016 followed by a decline in 2019—is a complex issue with multiple potential causes (Figure 2). These findings align with a previous study on breastfeeding practices, particularly, showing a decline in ever-breastfeeding and inconsistent trends in early initiation (38). The possible reasons might be demographic changes, dietary pattern changes and an increase in access to formula milk. Another possible explanation might be that public health interventions might have declined in intensity or quality after 2016, which could have contributed to the subsequent drop in exclusive or predominant breastfeeding practices (21, 39). However, predominant breastfeeding declined from 2000 to 2016 and subsequently increased in 2019. The study revealed no significant associations between these rates and the year of the survey, despite these fluctuations suggesting that these breastfeeding practices are not improving. These findings contrast with a study that concluded that breastfeeding practices improved in LMICs over the past decade despite regional variation (40). A possible explanation might be reflected in dietary pattern changes, increased access to formula milk, and demographic changes in recent years.

Except for the Gambella regional state of Ethiopia, which showed a different trend, the rates of exclusive and predominant breastfeeding across most Ethiopian regions fluctuated in a similar pattern from 2000 to 2019 (Figure 2). A possible explanation might be that mothers from Gambella might receive interventions of different intensities or qualities than mothers from other regions. Exclusive breastfeeding was higher in rural areas than in urban areas and declined ~2005, followed by an increase in 2016 and a decline again which is consistent with a systematic review and meta-analysis findings from Ghana (41). Higher exclusive breastfeeding rates in rural areas of Ethiopia compared to urban areas may be attributed to factors such as traditional postpartum care practices, limited access to commercial formula milk products, and lower socioeconomic status among rural mothers (41, 42). Similarly, higher predominant breastfeeding rates were observed in urban areas than in rural areas, where mothers who reside in urban areas could access formula milk and prefer complementary feeding earlier (43). To understand this, we need to consider various factors influencing breastfeeding practices in these settings.

The disparities in exclusive and predominant breastfeeding rates across different geographical and demographic groups in Ethiopia are consistent with global trends (40). These disparities highlight the complex interplay of factors influencing breastfeeding practices (39). Regional state, residence, and maternal religion were significantly associated with the likelihood of practicing exclusive and predominant breastfeeding (*p* < 0.01) (Table 2). For example, mothers residing in Amhara (AOR: 5.036), Oromia (AOR: 4.418), and former SNNP (AOR: 3.997) have significantly higher rates of exclusive breastfeeding than mothers residing in Somali, which is consistent with previous studies (44). These findings also reflected the rural-to-urban disparities. Higher rates of exclusive and predominant breastfeeding were observed among rural mothers than among urban mothers. These findings were consistent with previous findings (25, 44). These differences might be attributed to differences in socioeconomic status, stronger cultural norms supporting breastfeeding, and limited access to formula in rural areas (41, 42). Additionally, rural areas might have more entrenched cultural beliefs and practices supporting breastfeeding with extended family living arrangements, and larger families could provide more support for breastfeeding mothers (45). Another factor might be that the lower availability of formula in rural areas might encourage exclusive breastfeeding.

Mothers aged 25–29 years are less likely to exclusively or predominantly breastfeed than younger mothers aged 15–19 years are (*p* < 0.05). While specific studies focusing on this age group might be limited, general trends in breastfeeding and maternal age in which maternal age is significantly associated with exclusive breastfeeding have been explored in the literature (46). The possible explanations might be a greater likelihood of employment at the age of 25–29 years compared with younger mothers and lower family living arrangements and support from family. Differences in exclusive and predominant breastfeeding practices were observed among different religious groups. For example, Protestant and Orthodox mothers in Ethiopia have significantly higher rates of exclusive and predominant breastfeeding than Muslim mothers do (*p* < 0.001). Several studies have revealed that regions influence various health behaviors and breastfeeding practices (47–49). Potential reasons for religious differences in exclusive and predominant breastfeeding practices might be associated with religious beliefs and practices and the spatial residence of mothers. However, this highlights the need for additional qualitative studies.

The effect of a mother's education level on the likelihood of exclusive and predominant breastfeeding is not clear. Mothers with complete secondary education have a significantly lower prevalence of exclusive and predominant breastfeeding than mothers with no education, which seems counterintuitive. Educational attainment is among the predictors of breastfeeding (47). Conversely, some studies have reported lower breastfeeding rates among educated women, particularly in urban setting (17, 50). For example, analysis of the PMA2023 panel survey data (2021–2023) showed that less educated women in Ethiopia had higher rates of exclusive breastfeeding (50). The possible explanations might be that educated women are more likely to be employed, which can interfere with exclusive breastfeeding and have greater exposure to infant formula marketing.

In contrast to our hypotheses and those of previous studies, marital status (51, 52), employment (25), place of delivery (16, 44), antenatal care utilization (25, 44, 53) and postnatal care utilization (44, 53), and family size (25) were not significantly associated with the likelihood of exclusive and predominant breastfeeding. There might be possible explanations for these counterintuitive findings. The present study used nationally representative data combined from EDHS 2000–2019, whereas other studies were fragmented into different geographical locations. These differences might be due to the study design (sample size, data collection methods, and study period) and the unique characteristics of the national context.

### 4.1 The strengths and limitations of the study

The study used data from the Demographic and Health Surveys (EDHS) for Ethiopia from 2000 to 2019. The EDHS is a large-scale household survey that collects data on a variety of topics, including breastfeeding practices. This provides a strong foundation for the study. The study used a variety of statistical methods, including pooled multivariate logistic regression analysis, and prevalence odds ratios. This demonstrates a strong understanding of statistical methods and the ability to apply them to complex data.

This study has several considerable limitations. It is based on data from the EDHS, which is a cross-sectional survey. This means that the data can only show associations between factors and cannot be used to establish causality. The study did not control for some potential confounding factors, such as the wealth index, due to a lack of availability of data in all the EDHS datasets. The study did not include variables related to other potential factors that could influence breastfeeding practices, such as social support. Future research should address these limitations by using longitudinal data and collecting data that are more comprehensive on potential confounding factors.

## 5 Conclusion

Exclusive and predominant breastfeeding rates have varied across children aged <6 months in Ethiopia and have fluctuated inconsistently over the past two decades. These inconsistent trends in exclusive and predominant breastfeeding and a complex interplay of various factors suggest that previous policies and strategies have had limited success in achieving sustained improvements. The significant influence of regional, residential, and religious factors on the likelihood of these breastfeeding practices highlights the need for targeted and tailored public health interventions about exclusive and predominant breastfeeding. These findings suggest a need for further investigation and revisiting the current policies and implementation strategies for a more nuanced and targeted approach in future interventions. The policy and intervention strategies need to be revised through regular assessments and evaluation research that considers regional differences and sociocultural settings for equitable and sustained improvements in breastfeeding in Ethiopia. Further study is needed to explore the interplay between maternal age, employment status, and breastfeeding practices in more detail, investigating social support networks and childcare arrangements and the knowledge, attitudes, and beliefs about breastfeeding among different age groups of mothers to develop targeted interventions.

## Data Availability

Publicly available datasets were analyzed in this study. This data can be found here: http://www.dhsprogram.com/.
